# Posterior cruciate ligament tibial attachment sacrifice percentage is higher in cruciate-retaining total knee arthroplasty in patients with discoid lateral meniscus

**DOI:** 10.1186/s42836-024-00238-2

**Published:** 2024-04-03

**Authors:** Weiwei Xin, Yingjian Gao, Liangjun Zheng, Xinhua Qu, Bing Yue

**Affiliations:** Department of Orthopedics, School of Medicine, Renji Hospital, Shanghai Jiaotong University, Shanghai, 201112 China

**Keywords:** Posterior cruciate ligament, Cruciate-retaining total knee arthroplasty, Discoid lateral meniscus, Magnetic resonance imaging

## Abstract

**Background:**

The posterior cruciate ligament (PCL) attachment may be damaged in cruciate-retaining total knee arthroplasty (CR-TKA) using the complete resection for tibial preparation, and resection amount varies greatly among individuals. Discoid lateral meniscus (DLM) is one of the most common anatomic knee variants. This study aimed to evaluate the difference in PCL attachment sacrifice in CR-TKA between patients with and without DLM.

**Methods:**

Fifty-one knees in the study group (DLM group) were matched 1:1 to 51 control knees (non-DLM group) by age, sex, and maximum width of the tibial plateau. The percentage of the sacrificed PCL attachment and the morphological parameters of the tibial plateau were evaluated using magnetic resonance imaging (MRI) in a blind manner.

**Results:**

With a tibial cut simulated at a 0°, 3°, and 7° osteotomy slope, the mean PCL attachment resection percentages in the non-DLM group were 40.5%, 53.6%, and 72.6%, respectively. The corresponding resection percentages in the DLM group were 61.0% (*P* < 0.001), 73.3% (*P* < 0.001), and 85.7% (*P* < 0.001), respectively. The percentage of the minimum meniscus width to the maximum tibia width showed a weak positive correlation with the percentage of PCL attachment sacrifice.

**Conclusions:**

A significantly greater portion of PCL attachment was sacrificed in DLM patients undergoing CR-TKA using the complete proximal tibia resection. Attention should be paid to PCL attachment resection during CR-TKA in patients with DLM, and alternative techniques or prosthesis types should be considered.

**Graphical Abstract:**

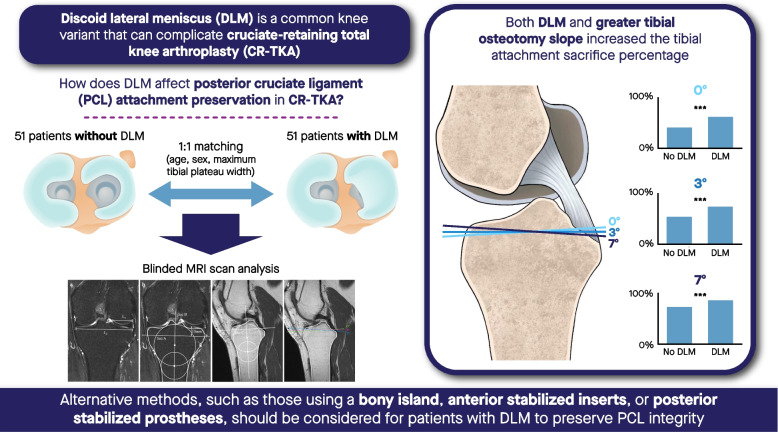

## Background

Cruciate-retaining (CR) prosthesis is a common type of total knee arthroplasty (TKA) prostheses. In CR-TKA, a bony island is often recommended to preserve the entire tibial attachment of the PCL. However, this small bony island tends to be fragile and is susceptible to fracture due to the excessive mechanical stress on the PCL attachment intraoperatively or with active movement postoperatively. Therefore, many surgeons prefer to completely resect the proximal tibia without preserving the bone island [1–3]. However, with this approach, the attachment of the PCL will be at least partially damaged. The impact of the tibial osteotomy level on the preservation of the PCL attachment varies with patients, owing to the individual variation in knee morphology [1–11].

Discoid lateral meniscus (DLM) is one of the most common anatomic knee variants, with an incidence standing at 3–5% in the United States and hovering at 15% in Asia [12]. It is a vestigial nuisance and troublemaker in the knee [13]. A higher prevalence of varus knee deformity and a higher prevalence of osteoarthritis (OA) have been reported in middle-aged patients with a torn DLM [14]. The effect of DLM on the preservation of the PCL attachment in CR-TKA using the complete proximal tibia resection has not been previously reported. This study aimed (1) to evaluate the difference in the percentage of PCL attachment sacrifice between DLM subjects and their non-DLM counterparts using magnetic resonance imaging (MRI), and (2) to assess morphological interaction factors other than the meniscus width in case a difference is found.

## Methods

### Patient selection

**Fig. 1:**
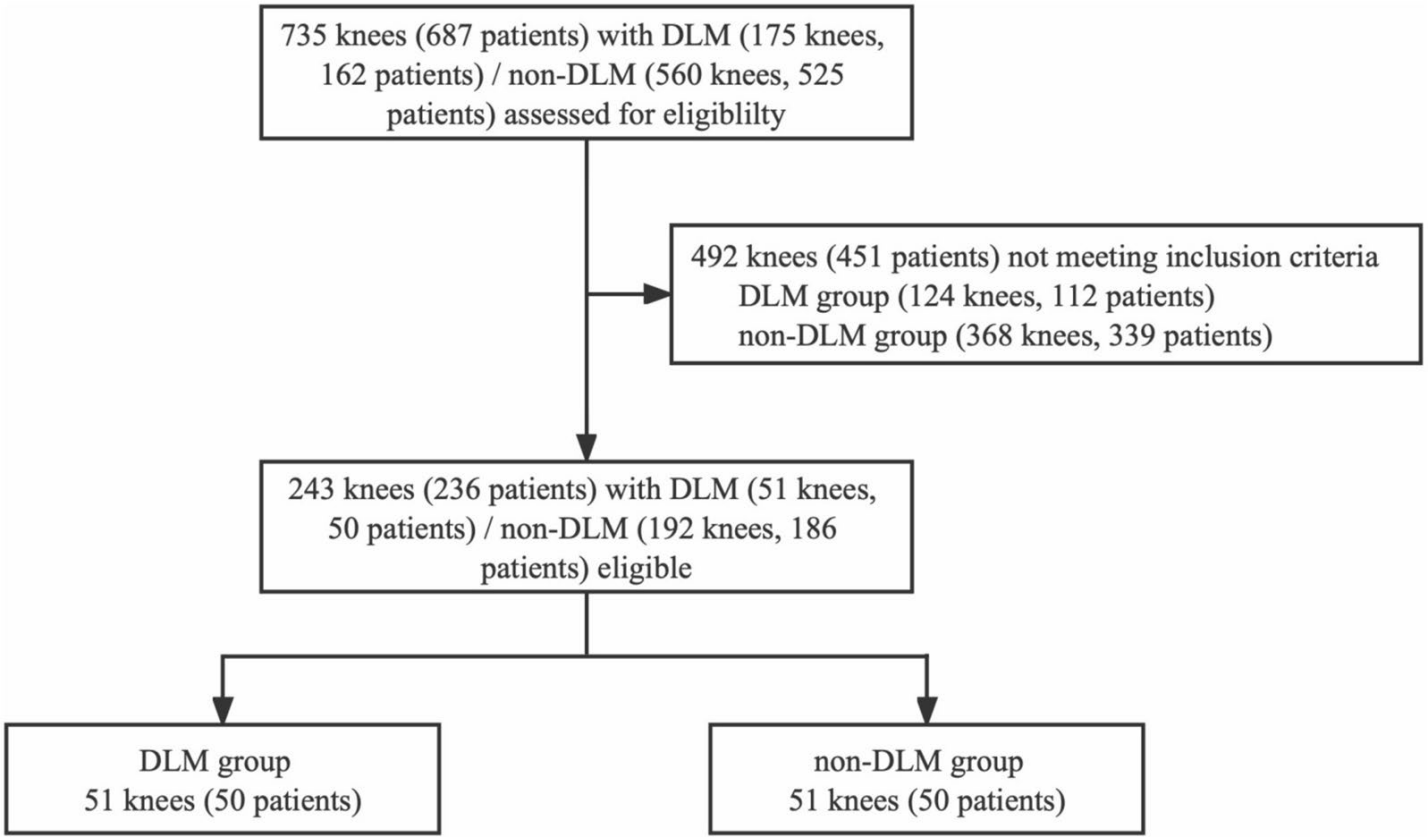
Flow diagram for the study. DLM = discoid lateral meniscus

**Fig. 2:**
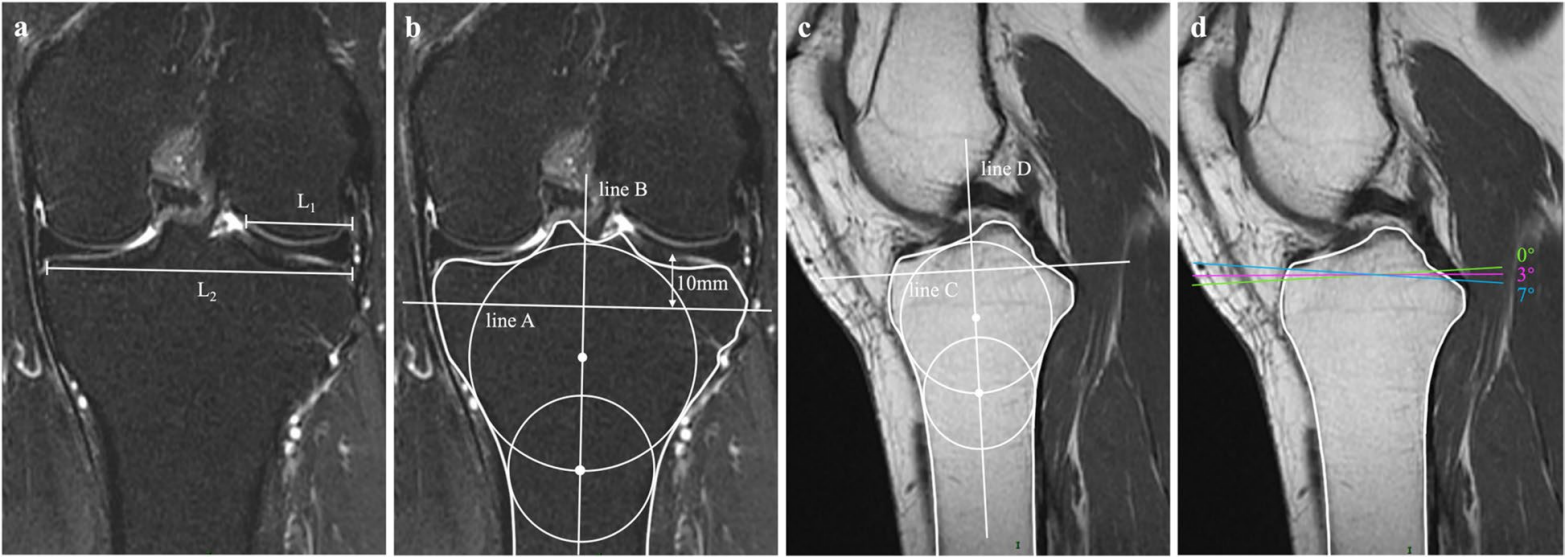
DLM diagnostic criteria and procedure for measuring the PCL attachment sacrifice percentage at different osteotomy slopes. **a** Diagnostic criteria of DLM: LMW% = L_1_ / L_2_*100%; **b** The first step was to define the osteotomy plane on the coronal slice; **c** The second step was to define the osteotomy plane at a 0° slope on the central sagittal slice; **d** Measuring the length of PCL attachment and the length of PCL attachment sacrificed at three osteotomy slopes. DLM = discoid lateral meniscus, LMW% = the percentage of the minimum meniscus width to the maximum tibia width, L_1_ = the minimum meniscus width, L_2_ = the maximum tibia width

This study was performed in line with the principles of the Declaration of Helsinki. Upon ethical approval by the institutional review board (LY2023-068-B), 175 consecutive knees clinically diagnosed with DLM from April 2021 to March 2022 were included in this retrospective investigation (Fig. 1). DLM was diagnosed when the percentage (LMW%) of the minimum meniscus width to the maximum width of the tibial plateau (WTP) was greater than 20% (Fig. 2a) [15]. Patients were excluded from the study if they met at least one of the following criteria: (1) age < 18 years; (2) skeletal immaturity; (3) PCL injury; (4) dysplasia of the knee; (5) proximal tibia fracture; (6) history of surgery in the proximal tibia; (7) severe misalignment of the proximal tibia; and (8) insufficient quality of MRI scans. A power analysis conducted in a pilot study with a tibial cut simulated at 0° showed that a minimum sample of 21 knees in each group was needed to achieve 90% power.

A total of 51 knees (50 patients) were included in the DLM group. The knees were matched in terms of age, sex, and WTP in a 1:1 ratio to 51 control knees (non-DLM group, 50 patients) diagnosed as non-DLM from January 2022 to March 2022 (Fig. 1). The foregoing exclusion criteria also applied to the non-DLM group. In total, 102 images from 62 women and 40 men were included in the study. A slightly more knees (55.9%) were left side and the average patient age was 46.2 (18–76) years old.

### MRI analysis

Magnetic Resonance Imaging (MRI) scans were acquired by using a 3.0-Tesla Signa HDXT scanner (GE Healthcare, Milwaukee, WI, USA) and a 3.0 T MR imaging system (Ingenia 3T; Philips Healthcare, Best, Netherlands). The MRI protocol included coronal T2-weighted, axial T2-weighted, and sagittal T2-weighted fat-suppression phases, as well as a sagittal T1-weighted phase. All MRI images were reviewed in a blind fashion by two senior orthopedic surgeons with expertise in joint surgery and sports medicine using the Carestream Vue Picture Archiving and Communication System (Vue PACS; Carestream Health Inc., Rochester, NY, USA). Based on the sagittal, coronal, and axial sections, measurements were conducted using a 4-step method. First, a simulated osteotomy plane was determined on the coronal slice that passed through the highest point of the articular cartilage on the sagittal slice located at the center of the lateral tibial plateau. A circle tangent to the proximal, lateral, and medial tibial borders was drawn. Subsequently, a second circle was drawn, with its center on the perimeter of the first circle and tangent to the lateral and medial tibial diaphyseal borders. A line (line A), representing the osteotomy plane on the coronal plane, was drawn perpendicular to the tibial coronal longitudinal axis (line B) which connected the two centers of the circles, and 10 mm caudally to the lateral highest point of the articular cartilage, as described previously (Fig. 2b) [10]. Second, a circle was drawn tangent to the proximal, anterior, and posterior tibial borders on the central sagittal slice, which consisted of the intercondylar eminence, the anterior and posterior tibial cortices appearing as a concave shape, and the tibial attachment of the PCL. A second circle was drawn tangent to the anterior and posterior tibial diaphyseal borders, with its center on the perimeter of the first circle. A line (line C), representing the 0° osteotomy slope on the sagittal plane, was drawn perpendicular to the tibial sagittal longitudinal axis (line D), which connected the two centers of the circles (Fig. 2c) [16]. Third, the osteotomy plane was determined on the sagittal plane based on lines A and C. The posterior slope of the simulated sagittal osteotomy plane was set relative to line C at 0°, 3°, and 7° (Fig. 2d) to cover the range of the most used implants [2, 8, 10]. Fourth, the proximal and distal points of the tibial attachment of the PCL were determined on the sagittal slice containing the widest tibial attachment of the PCL [8]. The intersection of the resection plane and the tibial PCL attachment was marked. The percentage of sacrificed PCL attachment was calculated by dividing the amount of PCL resection at each osteotomy slope by the length of the PCL. The medial posterior tibial slope (MPTS) was defined as the angle between line C and the tangent to the medial cartilage surface of the tibial plateau on a sagittal slice located at the center of the medial tibial plateau. The lateral posterior tibial slope (LPTS) was defined as the angle between line C and the tangent to the lateral cartilage surface of the tibial plateau on a sagittal slice located at the center of the lateral tibial plateau [16]. Medial proximal tibial angle (MPTA) referred to the medial angle between a line joining the peak points on the medial and lateral aspects of the plateau and Line B.

### Reliability evaluation

Intra-class correlation coefficients were calculated by randomly selecting 20 knees (10 in the DLM group and 10 in the non-DLM group). Two observers separately evaluated the MRI images. In addition, one orthopedic surgeon performed two measurements, being one month apart. The intra-observer repeatability and inter-observer reliability were excellent (>0.9) and good (>0.8) respectively, for measuring the parameters and the percentage of the PCL sacrificed at each osteotomy slope.

### Statistical analysis

Analyses were conducted using RStudio (Integrated Development for R. RStudio, Inc., Boston, MA, USA). Categorical variables were presented as numbers with percentages and compared by using Pearson’s chi-square test or Fisher’s exact test. Continuous variables were expressed as means with ranges and compared using the student’s *t*-test or Wilcoxon rank sum test. Correlation analyses were performed to determine the strength of the linear relationships between the percentage of sacrificed PCL attachment and morphological parameters of the knee joint. For all statistical analyses, a *P* value of <0.05 was considered statistically significant.

## Results

For the pooled population, the WTP was 69.5 (60.8–80.9) mm. Lateral meniscal injury, medial meniscal injury, ACL injury, osteochondral lesion of the lateral plateau, and osteochondral lesion of the medial plateau were present in 20.6%, 27.5%, 6.9%, 2.9%, and 7.8% of images, respectively. The average length of the PCL attachment in the sagittal plane on MRI was 10.9 (6.7–14.9) mm. A tibial cut with a 0° slope caused a sacrifice of 50.7% (0–100%) of PCL attachment. This rose to 63.4% (0–100%) and 79.1% (17.5–100%) respectively, when a 3° or 7° tibial cut slope was simulated.

**Fig. 3:**
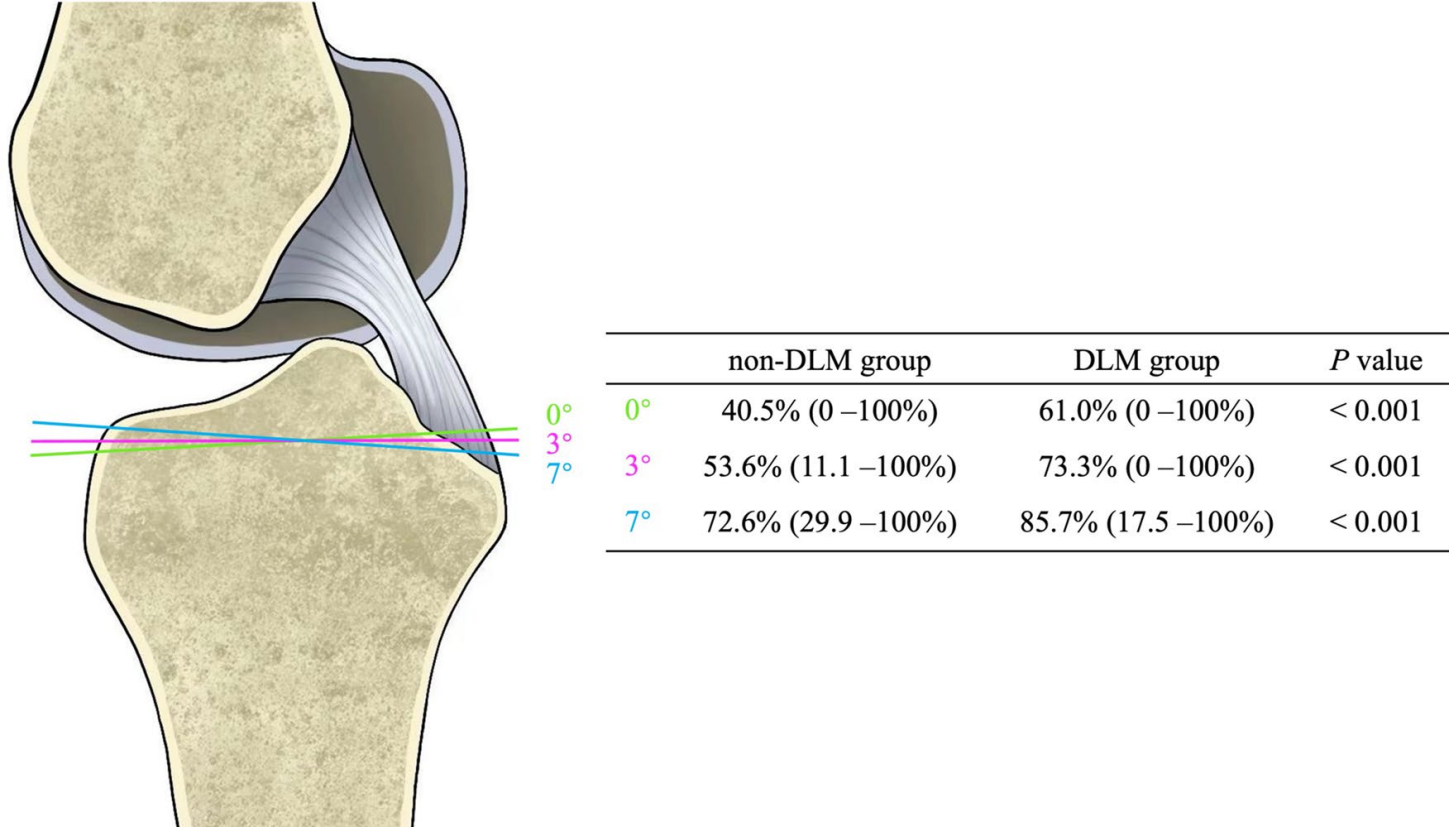
PCL attachment sacrifice percentage at different osteotomy slopes in non-DLM and DLM groups. DLM = discoid lateral meniscus

**Fig. 4:**
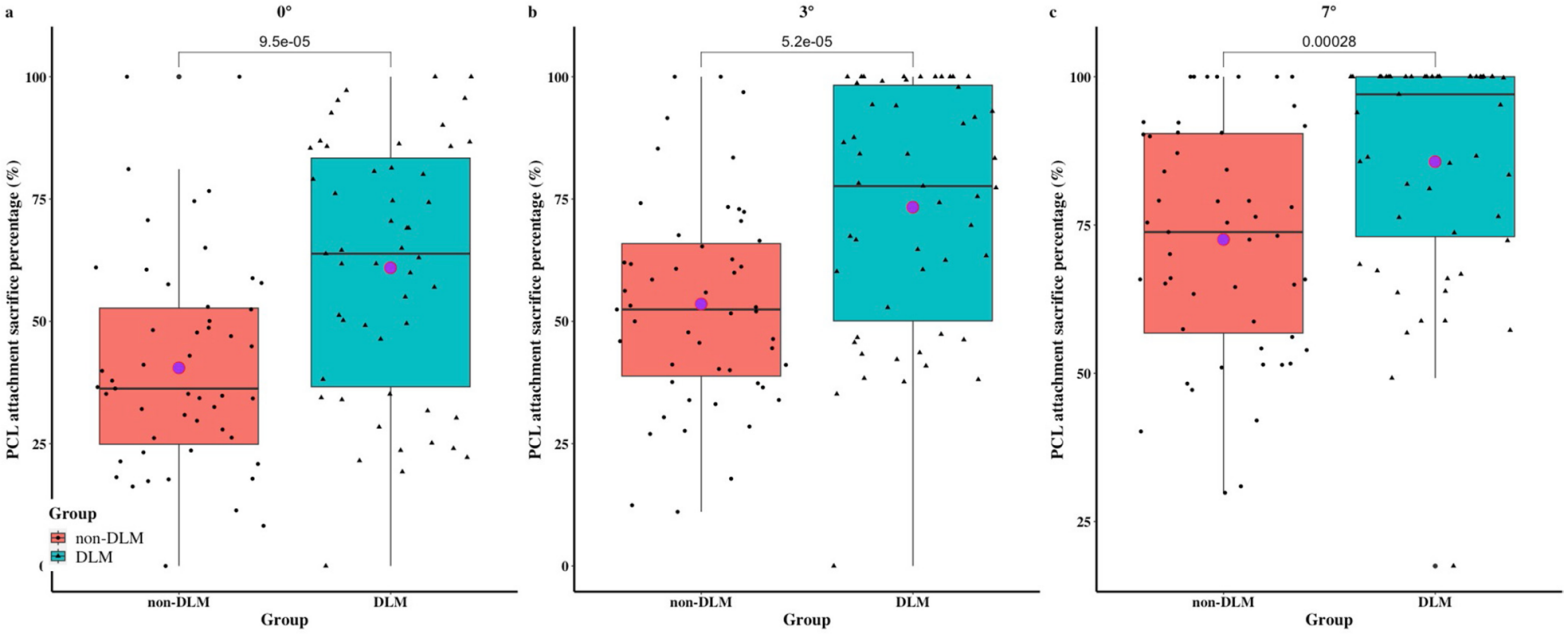
PCL attachment sacrifice percentage in non-DLM and DLM groups. **a** With a tibial cut simulated at a 0° slope, 40.5% (0–100%) vs. 61.0% (0–100%), *P* < 0.001; **b** With a 3° slope, 53.6% (11.1–100%) vs. 73.3% (0–100%), *P* < 0.001; **c** With a 7° slope, 72.6% (29.9–100%) vs. 85.7% (17.5–100%), *P* < 0.001. DLM = discoid lateral meniscus

The data for each group are summarized in Table 1. At the osteotomy slopes of 0°, 3°, and 7°, 61.0% (0–100%), 73.3% (0–100%), and 85.7% (17.5–100%) of the tibial PCL attachments were resected in the DLM group, against 40.5% (0–100%, *P* < 0.001), 53.6% (11.1–100%, *P* < 0.001), and 72.6% (29.9–100%, *P* < 0.001) in the non-DLM group, respectively (Figs. 3 and 4).

**Table 1:** Descriptive statistics of DLM group and non-DLM group

	**non-DLM Group**	**DLM Group**	***P***
Age (yr)	45.3 (18–76)	47.0 (21–70)	0.534
Women^a^	31 (60.8)	31 (60.8)	1
Left side^a^	25 (49)	32 (62.7)	0.163
WTP (mm)	69.5 (60.8–80.7)	69.5 (62.0–80.9)	0.996
LMW%	15.6 (9.1–19.9)	29.5 (20.2–43.3)	<0.001
Length of PCL (mm)	10.7 (6.7–14.9)	11.2 (7.5–14.7)	0.112
LPTS (°)	5.2 (-0.5–14.0)	6.4 (1.4–14.4)	0.103
MPTS (°)	6.3 (0.7–12.9)	7.3 (0.3–13.0)	0.044
MPTA (°)	88.5	87.9	0.048
Disease^a^			
Lateral meniscus injury	4 (7.8)	17 (33.3)	0.001
Medial meniscus injury	14 (27.5)	14 (27.5)	1
ACL injury	3 (5.9)	4 (7.8)	1
Osteochondral lesion of lateral plateau	1 (2.0)	2 (3.9)	0.269
Osteochondral lesion of medial plateau	2 (3.9)	6 (11.8)	1

*DLM* discoid lateral meniscus, *LMW%* the percentage of the minimum meniscus width to the maximum tibia width, *WTP* maximum width of tibial plateau, *PCL* posterior cruciate ligament, *LPTS* lateral posterior tibial slope, *MPTS* medial posterior tibial slope, *MPTA* medial proximal tibial angle

^a^The values are given as the number of knees, with the percentage in parentheses

**Fig. 5:**
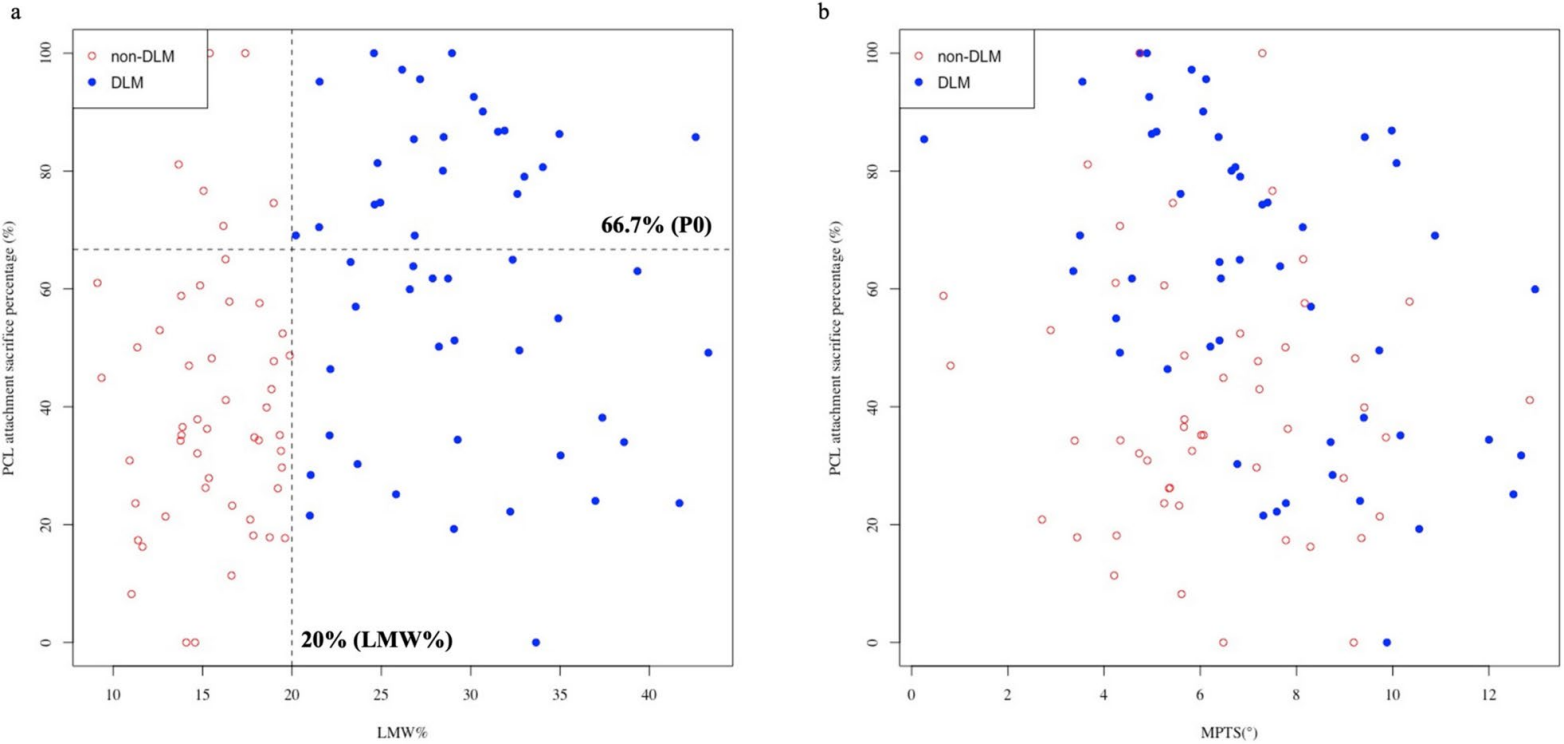
The correlation between PCL attachment sacrifice percentage and morphological feature. **a** Graph showing LMW% and the percentage of PCL attachment sacrificed with a tibial cut simulated at a 0° slope. The two dashed lines represented the cut-off of DLM and 66.7% of PCL sacrificed; **b** Graph showing MPTS and the percentage of PCL attachment sacrificed with a tibial cut simulated at a 0° slope. LMW% = the percentage of the minimum meniscus width to the maximum tibia width, P0 = the percentage of PCL attachment sacrificed with a tibial cut simulated at a 0° slope, MPTS = medial posterior tibial slope, DLM = discoid lateral meniscus

To provide added insight into the differences in the percentage of sacrificed PCL attachment between the two groups, the LMW% and the percentage of PCL attachment sacrifice at 0° were plotted on the same graph (Fig. 5a). The graph was then divided into four quadrants by two lines representing the cut-off of DLM and 66.7% of PCL attachment sacrifice. The latter indicated a complete removal of the anterolateral (AL) bundle of PCL [5]. The data points, for a substantial proportion of the DLM subjects (45.1%), were located in the upper quadrants, against 11.8% of the non-DLM subjects, RR = 3.833 (1.789–8.605), *P* < 0.001. Similar results were yielded for simulated osteotomy at 3° [60.8% vs. 23.5%, RR = 2.583 (1.547–4.508), *P* < 0.001] and 7° [82.4% vs. 56.9%, RR = 1.448 (1.119–1.944), *P* = 0.009].

For the pooled population, the correlation between the percentage of PCL attachment sacrifice and LMW%, and between the percentage of PCL attachment sacrifice and MPTS was poor, as evidenced by the correlation coefficients being 0.29 and -0.21 with a 0° slope (Fig. 5a, b), 0.29 and -0.22 with a 3° slope, and 0.22 and -0.24 with a 7° slope, respectively. No other linear relationship was found between the percentage of PCL attachment sacrifice and morphological parameters. The plots, constructed with use of the MPTS and the percentage of PCL attachment sacrifice with a 0° slope, showed that the data points of the DLM subjects were skewed to the upper right quadrant compared to those of the non-DLM subjects (Fig. 5b).

## Discussion

Preservation of the PCL attachment is critical for CR-TKA because damage to the footprint of the PCL in CR-TKA could jeopardize the functional integrity of the PCL over time. The main finding of this study was that about 20% more PCL fibers were sacrificed in the DLM group compared to the non-DLM group at a 0° osteotomy slope (61.0% vs. 40.5%) and at a 3° osteotomy slope (73.3% vs. 53.6%). The difference was 85.7% vs. 72.6% at a 7° osteotomy slope. A significantly greater proportion of subjects with DLM had over 66.7% of sacrificed PCL at a 0° slope (45.1% vs. 11.8%, RR = 3.833), a 3° slope (60.8% vs. 23.5%, RR = 2.583), and a 7° slope (82.4% vs. 56.9%, RR = 1.448). LMW% showed a weak positive correlation with the percentage of PCL attachment sacrifice. However, MPTS exhibited a poor negative correlation with the percentage of PCL attachment sacrifice.

Several studies have evaluated the sacrificed PCL attachment during CR-TKA [1–11]. Shannon et al. [3] found that a portion, or all, of PCL tibial attachment, was sacrificed in more than 75% of cases when using CR prostheses. A number of quantitative studies have shown that 35% to 75.7% of PCL attachment was sacrificed, simulating tibial osteotomies with a depth of 10 mm and 0° to 7° slopes on MRI [5, 7, 8, 10]. In a cadaveric study, 68.8 ± 15.3% of the PCL attachment was removed with a tibial cut at a depth of 9 mm at a 3° posterior slope [11]. These previous observations are consistent with our results. However, to our knowledge, there have been no studies comparing the difference in PCL attachment damage between DLM and non-DLM subjects. Given the configuration and dimensions of the AL and posteromedial (PM) bundles of the PCL, resection of the anterior two-thirds of the PCL tibial attachment resulted in the complete removal of the AL bundle [5]. Our data indicated that DLM is a risk factor for the removal of the AL bundle of PCL at a 0°, 3°, and 7° osteotomy slope (Fig. 5a). In the non-DLM group, osteotomy slopes >3° also caused complete removal of the AL bundle (Fig. 4). Because the AL bundle is stretched during flexion to stabilize the knee, whereas the PM bundle is tightened during extension, iatrogenic PCL injury is a potential cause of late flexion instability after CR-TKA and may lead to future revisions [5]. Therefore, alternative TKA techniques, such as those using a bony island, anterior stabilized (AS) inserts, and PS prostheses, should be considered for patients with DLM.

As measured with MRI, the length of the PCL tibial footprint was found to range from 11.3 mm to 19.0 mm [1, 5, 10]. The size of the PCL tibial footprint correlated with the subject’s sex, height, and dimensions of the tibial plateau [2, 4]. As the thickness of the lateral tibial plateau resection is fixed at 10 mm, the thickness of the posterior part of the resected fragment at a given osteotomy slope may vary, depending on patient features. Likewise, in our study, the PCL attachment sacrifice varied with sex and age (data not presented). Therefore, to avoid bias, we matched patients by sex, age, and body size. In this study, the length of PCL attachment was 10.9 mm, which was similar to the result of 11.3 mm in a previous study conducted in Japanese patients [1]. Some other factors may also affect the PCL attachment sacrifice, such as the resection reference, tibial osteotomy depth, and slope [4, 5]. Nevertheless, exploring the impact of these factors on PCL attachment sacrifice was beyond the scope of this study. To avoid bias due to such factors, we performed simulated osteotomies in the two groups using the same routine surgical strategy described in previous studies [4, 5, 10, 16].

In this study, some tibial parameters were also evaluated, including MPTS, LPTS, MPTA, and LMW%. The posterior slopes of the lateral and medial tibial plateau in this study were 5.8° and 6.8° respectively, consistent with prior reports [17, 18]. Sessa *et al*. reported a weak negative correlation between the amount of PCL lost and the degree of posterior inclination of the tibial plateau [8]. Our data identified a similar correlation between the MPTS and the PCL attachment sacrifice in the pooled population. LMW% showed a weak positive correlation with the PCL attachment sacrifice. In Figure 5b, the data points of DLM skewed to the upper right quadrant, indicating that the DLM group had a larger MPTS, but a greater PCL attachment sacrifice. This reverse correlation implied that DLM is an independent contributor to PCL attachment sacrifice, besides posterior tibial slope. Whereas no linear correlation existed between the LPTS and the PCL attachment sacrifice, and between MPTA and the PCL attachment sacrifice. Previous studies have found that DLM presented with a cupping of the lateral tibial plateau, obliquity of the lateral tibial articular surface, and hypoplasia of the lateral intercondylar spine [19, 20], which might be potential reasons for the DLM affecting the PCL attachment. Further in-depth studies are needed to explore these relationships.

Our study has some limitations. First, it was based on imaging simulation, which may not exactly replicate actual surgical outcomes. While it would be a better approach, a cadaveric study usually includes a limited sample size, especially for DLM cases, which may not be representative of the wide range of tibial morphologies in the general population. Imaging simulation measurement is one of the most commonly used methods for preoperative planning. The radiological analysis methods in this study have been used to assess the risk of sacrificing the PCL in CR-TKA in multiple studies [5, 6, 9, 10]. Second, linear measurements of PCL attachment are not fully representative of the PCL attachment, because the footprint is not symmetrical. Future three-dimensional model measurements based on MRI can provide more visual and detailed information.

## Conclusions

The complete resection technique for tibial preparation in CR-TKA may result in damage or removal of a significant part of the tibial PCL attachment, particularly for patients with DLM. Therefore, alternative techniques or prostheses are recommended for these patients.

## Data Availability

The datasets generated and analyzed during the current study are available from the corresponding author on reasonable request.

## Abbreviations

AL: anterolateral
AS: anterior stabilized
CR: cruciate-retaining
CR-TKA: cruciate-retaining total knee arthroplasty
DLM: discoid lateral meniscus
LMW%: percentage of the minimum meniscus width to the maximum width of the tibial plateau
LPTS: lateral posterior tibial slope
MPTA: medial proximal tibial angle
MPTS: medial posterior tibial slope
MRI: magnetic resonance imaging
PCL: posterior cruciate ligament
PM: posteromedial
PS: Posterior substituting
TKA: total knee arthroplasty
WTP: maximum width of the tibial plateau
